# Observational Burden of Illness Study in Patients With Crohn’s Disease With and Without Perianal Fistulas in the United States

**DOI:** 10.1016/j.gastha.2023.08.011

**Published:** 2023-08-24

**Authors:** Jeanne Jiang, Susan E. Cazzetta, Amod Athavale, Maja Kuharic, Tao Fan, Abigail Silber, Vijay Abilash, Nandini Hadker, Emily Sharpe, Pradeep P. Nazarey

**Affiliations:** 1 Takeda Pharmaceuticals USA, Inc., Lexington, Massachusetts; 2 Trinity Life Sciences, Waltham, Massachusetts; 3 Department of Pharmacy Systems, Outcomes and Policy, College of Pharmacy, University of Illinois, Chicago, Illinois

**Keywords:** Crohn’s Perianal Fistulas, Fecal Incontinence, Health-Related Quality of Life, Symptom Burden

## Abstract

**Background and Aims:**

This study compared disease burden, experiences, and health-related quality of life (HRQoL) between patients with Crohn’s perianal fistulas (CPFs) and those with Crohn’s disease (CD) without perianal fistulas (PFs; non-PF CD).

**Methods:**

This cross-sectional, observational study was conducted in 3 cohorts of US patients aged 18–89 years with self-reported, physician-diagnosed CD: (1) non-PF CD; (2) CPF without PF-related surgery; and (3) CPF with PF-related surgery. Data on medical and surgical interventions, CD-specific symptoms, HRQoL (assessed using the Short Inflammatory Bowel Disease and 5-dimension EuroQol questionnaires), and fecal incontinence (assessed using Revised Faecal Incontinence Scale and Fecal Incontinence Quality of Life questionnaires) were collected via a web-enabled questionnaire.

**Results:**

In total, 403 patients with CD completed the questionnaire (non-PF CD, n = 300; CPF without surgery, n = 51; CPF with surgery, n = 52). A high symptom burden was seen across cohorts. More patients with CPF underwent ≥1 CD-related surgery and experienced ≥1 failure of CD-related surgery (79% and 20%) vs non-PF CD (53% and 9%; *P* < .001). Overall HRQoL outcomes were worse for patients with CPF vs non-PF CD, with significantly worse Short Inflammatory Bowel Disease and 5-dimension EuroQol questionnaire scores for those without PF-related surgery (*P* < .01). Across all cohorts, 58% of patients reported experiencing fecal incontinence, which had a greater negative impact (higher Revised Faecal Incontinence Scale scores; lower Fecal Incontinence Quality of Life scores) in patients with CPF vs non-PF CD.

**Conclusion:**

Patients with CPF experience substantial HRQoL burden, reflecting the impact of symptoms and medical/surgical interventions. These results may help to inform comprehensive care strategies to improve patient HRQoL.

## Introduction

Crohn’s disease (CD) is a chronic inflammatory condition affecting the gastrointestinal tract and is complicated by the development of fistulas in up to 50% of patients.^1^^,^^2^ The cumulative incidence of perianal fistulas (PFs) in patients with CD is estimated to be 12%–15%, 21%–23%, and 26%–28% over 5, 10, and 20 years, respectively.^2^, ^3^, ^4^, ^5^ PFs are considered a marker of more severe CD,^6^ with common symptoms including pain (perianal and pelvic), swelling, perianal lumps, intermittent purulent perianal drainage, and fecal incontinence.^1^

There is currently no standard of care for disease management of Crohn’s perianal fistulas (CPF) and different approaches may be taken by gastroenterologists and colorectal surgeons. The recommendations provided by respective organizations, such as the American Gastroenterological Association, the American College of Gastroenterology, and the American Society of Colon and Rectal Surgeons, reflect the different roles of gastroenterologists and colorectal surgeons.^7^, ^8^, ^9^

The recommendations from the American Gastroenterological Association and American College of Gastroenterology focus on the role of medical therapy. Medical therapy is the mainstay of CPF treatment because control of luminal disease is critical for fistula healing, and the use of biologics (with or without antibiotics for induction of fistula treatment) is recommended.^9^ Additionally, the American College of Gastroenterology advocates the use of a combination of medical and surgical interventions for symptomatic CPF.^8^ The American Society of Colon and Rectal Surgeons guidelines focus primarily on surgical intervention for symptomatic CPF, with fistulotomy recommended for the treatment of carefully selected patients with symptomatic, simple, low (ie, less than 30% involvement of the external anal sphincter) CPF. For complex CPF, draining setons, mucosal advancement flap surgery, and ligation of the intersphincteric tract may be used, while those with uncontrolled symptoms may require fecal diversion or proctectomy.^7^

Ultimately, the main goal of therapy is to achieve and maintain remission of the fistulas while preserving sphincter function.^6^, ^7^, ^8^ However, many patients do not achieve adequate responses to available medical therapy or will subsequently lose response to biologic treatment.^10^^,^^11^ Furthermore, despite advances in surgical interventions over the past few decades, many patients experience failures and recurrence or persistence.^11^^,^^12^

Besides representing a substantial treatment burden for patients, CPF can significantly impair patients’ health-related quality of life (HRQoL) owing to physical symptoms and impaired social and emotional well-being.^1^^,^^13^ However, published studies on the clinical and humanistic burden in patients with CPF are limited. This study was conducted to gain a greater understanding of the burden of illness in patients with CD with and without PF, including symptom burden and its impact on HRQoL. In patients with CPF, the impact of PF-related surgery on burden of illness was also assessed.

## Materials and Methods

### Study Design

This cross-sectional, observational burden of illness study was conducted via a web-enabled questionnaire in patients with CD, with and without PF, in the United States. The primary objective of this study was to compare HRQoL, experiences, and perceptions between patients with CPF and non-PF CD using patient-reported outcome measures. The secondary objective was to compare the primary outcome measures among patients with CPF who received pharmacotherapy and/or a seton placement (but no other PF-related surgery) and those with CPF who had undergone PF-related surgery (with or without pharmacotherapy) in the past 12 months. Patients were asked to identify any PF-related surgeries they had had in the past 12 months from a list that included seton placement and/or removal, endorectal/anal advancement flap, fibrin glue, anal fistula plug, fistulectomy/fistulotomy, colectomy, ileostomy, colostomy, proctocolectomy, ligation of the intersphincteric tract, Martius flap, and rectovaginal fistula repair. Diagnostic procedures (eg, examination under anesthesia) were not included in the list of PF-related surgeries.

### Ethics

This study was conducted in accordance with the current version of the Declaration of Helsinki, Good Pharmacoepidemiology Practices, International Society for Pharmacoepidemiology Good Pharmacoepidemiology Practices guidelines, and any local regulations. Special attention was paid to data protection in line with the United States Code of Federal Regulations. The study was approved by an institutional review board to ensure compliance with all ethical standards.

### Patient Recruitment

Recruitment was undertaken by Dynata LLC (NY, USA). Patients were invited to participate in the study based on self-reported profile data for CD as part of Dynata’s panel of patients. Given the expected challenges with recruiting patients with CPF and the exploratory nature of this study, a sample of approximately 100 patients with CPF was deemed feasible and a 1:3 ratio was used to recruit the comparator cohort of patients with non-PF CD, resulting in a sample of 300 patients. Thus, a total study population of 400 patients with CD was the recruitment goal.

Inclusion criteria were age ≥18 years and ≤89 years at the time of consent, self-reported physician-diagnosed CD, and treatment for PF in the past 12 months (CPF cohorts), or never experienced PF (non-PF CD cohort). Patients diagnosed with ulcerative colitis were excluded from the study.

Patients were recruited into 1 of 3 cohorts: cohort 1, patients with non-PF CD; cohort 2, patients with CPF who received pharmacotherapy and/or seton placement(s) for PF in the previous 12 months, but no other PF-related surgery; and cohort 3, patients with CPF who underwent PF-related surgery with or without pharmacotherapy and seton placement in the previous 12 months.

Prior to completion of the web-enabled questionnaire, cognitive interviews (60 minutes) were conducted via telephone, during which the interviewer hosted the web-enabled questionnaire on their computer and shared their screen with the respondent via a screensharing software. These interviews were conducted to assess comprehension of the questions as intended and to pinpoint any potential sources of response error.

### Study Questionnaire

The 45-minute web-enabled questionnaire was administered via an Internet link hosted by Dynata LLC and included topics on demographics, patient diagnosis and symptom/treatment experience, and patient-reported outcome measures.

CD-specific symptom severity in the past 12 months was scored on a scale of 1–7, in which a higher score indicated a greater severity. The frequency of CD-specific symptoms in the past 12 months were reported as “every other month,” “once per month,” “every other week,” “once per week,” “twice per week,” or “every other day.”

All patients completed the Short Inflammatory Bowel Disease Questionnaire (SIBDQ)^14^ and the 5-dimension EuroQol questionnaire (EQ-5D).^15^ The SIBDQ is a validated inflammatory bowel disease-specific tool measuring HRQoL across 4 domains (bowel symptoms, systemic symptoms, social function, and emotional health) consisting of 10 items in total. The scores for each of the 4 domains were summed (a summed score difference of ≥9 points is considered to be clinically significant), then divided by 10 to achieve a mean score ranging from 1 (poor HRQoL) to 7 (optimal HRQoL), based on patient experience over a 2-week recall period.^14^ The 5-level EQ-5D (EQ-5D-5L) is a disease nonspecific instrument that consists of the following 2 parts: the EQ-5D-5L descriptive system and the EQ visual analog scale. The descriptive system assesses 5 dimensions (mobility, self-care, usual activities, pain/discomfort, and anxiety/depression), each with 5 levels (no, slight, moderate, severe, and extreme problems). Health state index scores were calculated from individual health profiles using values from the United States general population^16^ ranging from <0 (in which 0 is the value of a health state equivalent to death and negative values represent a health state worse than death) to 1 (the value of full health), with higher scores indicating higher health utility. The EQ visual analog scale was used to assess patients’ current self-rated health on a vertical scale ranging from 0 (worst imaginable health state) to 100 (best imaginable health state).

Patients who reported fecal incontinence additionally completed the Revised Faecal Incontinence Scale (RFIS)^17^ and the Fecal Incontinence Quality of Life questionnaire (FIQL).^18^ The RFIS consists of 5 items related to the effects of fecal incontinence and leakage on a person’s lifestyle and 2 additional items related to fecal incontinence associated with urge and undergarment soiling. Scoring is conducted by summing the scores assigned to each item, with the overall range being 0–20 (<4, no fecal incontinence or very mild; 4–6, mild; 7–12, moderate; ≥13, severe). The FIQL comprises 29 items forming 4 domains: lifestyle (10 items), coping/behavior (9 items), depression/self-perception (7 items), and embarrassment (3 items). The scale has a range of 0–5 for each item; lower scores indicate a worse quality of life (scoring is for domains only, no overall score is calculated).

### Statistical Analysis

Descriptive statistics were used to compare the outcome variables for each cohort and *P* values were calculated using t-tests. Statistical comparisons were assessed at an α level of 0.05, and multiple comparisons adjustment was applied when comparing >2 groups. Metrics captured on linear numeric scales with ≥7 points were considered continuous for the purposes of these analyses.

Generalized linear models were used to compare responses from patients with CPF (cohorts 2 and 3) and non-PF CD (cohort 1), with potential confounders (with a *P* value of .2 for variable retention) included as covariates in multivariable analyses to control for their effects.

Data were checked for potential outliers (prespecified as ≥3 standard deviation units for continuous variables). Straight liners (respondents who made the same selection across questions) were excluded as part of the data cleaning process.

## Results

### Patients

In total, 403 patients with CD completed the questionnaire from July to September 2020: n = 300 with non-PF CD (cohort 1) and n = 103 with CPF (without PF-related surgery [cohort 2], n = 51; with PF-related surgery [cohort 3], n = 52). Of those patients who accessed the questionnaire (n = 598), 131 did not pass data quality checks or were excluded owing to the patient quota being reached. The abandonment rate (the proportion of patients who accessed the questionnaire but did not complete it) was 64/598 (11%). The abandonment rate was slightly higher (22%) among patients with CPF who had surgery (cohort 3) compared with patients in other cohorts (9%).

As prespecified, 1 outlier and 1 straight liner were identified and removed prior to analysis (both in cohort 1). Demographics were broadly similar across the 3 cohorts (Table), although patients with non-PF CD were slightly older (mean [standard deviation] age 47 [16.4] years) compared with patients with CPF without surgery (40 [12.2] years) and CPF with surgery (39 [13.1] years).

**Table:** Demographics and Disease Characteristics in Patients With Non-PF CD and CPF With or Without PF-Related Surgery

Characteristic	Cohort 1: Non-PF CD (n = 300)	CPF			*P* Values (cohort 1 vs cohorts 2 + 3)
		Cohort 2: CPF without surgery (n = 51)^a^	Cohort 3: CPF with surgery (n = 52)	Cohorts 2 + 3: All CPF (n = 103)	
Age, y, mean (SD)	47 (16.4)	40 (12.2)	39 (13.1)	40 (12.6)	<.001
Sex, n (%)					
Male	127 (42.3)	26 (51.0)	29 (55.8)	55 (53.4)	.052
Female	173 (57.7)	25 (49.0)	23 (44.2)	48 (46.6)	.052
Ethnicity, n (%)					
Non-Hispanic White	222 (74.0)	33 (64.7)	35 (67.3)	68 (66.0)	.120
Hispanic or Latino	57 (19.0)	15 (29.4)	9 (17.3)	24 (23.3)	.347
Non-Hispanic Black or African American	17 (5.7)	2 (3.9)	6 (11.5)	8 (7.8)	.446
Native American or American Indian	0 (0.0)	0 (0.0)	0 (0.0)	0 (0.0)	NA
Asian/Pacific Islander	1 (0.3)	1 (2.0)	1 (1.9)	2 (1.9)	.101
Other	3 (1.0)	0 (0.0)	1 (1.9)	1 (1.0)	.979
Current medication use, n (%)					
No medication	64 (21.3)	3 (5.9)	7 (13.5)	10 (9.7)	.008
1 therapy	189 (63.0)	34 (66.7)	31 (59.6)	65 (63.1)	.928
2 therapies	37 (12.3)	6 (11.8)	8 (15.4)	14 (13.6)	.765
≥3 therapies	10 (3.3)	8 (15.7)	6 (11.5)	14 (13.6)	<.001
Currently used medications, n (%)					
Biologics	128 (42.7)	29 (56.9)	31 (59.6)	60 (58.3)	.008
Immunosuppressants/immunomodulators	44 (14.7)	13 (25.5)	11 (21.2)	24 (23.3)	.029
Anti-inflammatory agents	62 (20.7)	13 (25.5)	11 (21.2)	24 (23.3)	.471
Other	53 (17.7)	14 (27.5)	9 (17.3)	23 (22.3)	.226
Diagnosing physician for CD, n (%)					
Gastroenterologist	254 (84.7)	48 (94.1)	46 (88.5)	94 (91.3)	.093
Primary care physician	31 (10.3)	3 (5.9)	4 (7.7)	7 (6.8)	.289
Other	15 (5.0)	0 (0.0)	2 (3.8)	2 (1.9)	.183
Primary physician for CD, n (%)					
Gastroenterologist	251 (83.7)	44 (86.3)	43 (82.7)	87 (84.5)	.998
Primary care physician	41 (13.7)	5 (9.8)	6 (11.5)	11 (10.7)	.580
Surgeon	3 (1.0)	2 (3.9)	3 (5.8)	5 (4.9)	.016
CD severity (physician-assessed), n (%)					
Mild	53 (17.7)	6 (11.8)	7 (13.5)	13 (12.6)	.233
Moderate	191 (63.7)	31 (60.8)	28 (53.8)	59 (57.3)	.249
Severe	39 (13.0)	13 (25.5)	16 (30.8)	29 (28.2)	<.001
Unknown/unsure	17 (5.7)	1 (2.0)	1 (1.9)	2 (1.9)	.124
Recent CD flare, yes, n (%)	148 (49.3)	34 (66.7)	30 (57.7)	64 (62.1)	.248
CD complication frequency, mean (SD)	5.8 (4.1)	9.6 (3.9)	9.4 (4.4)	9.5 (4.1)	<.001
0–1 complication, n (%)	31 (10.3)	1 (2.0)	0 (0.0)	1 (1.0)	.002
2–5 complications, n (%)	147 (49.0)	7 (13.7)	9 (17.3)	16 (15.5)	<.001
>5 complications, n (%)	122 (40.7)	43 (84.3)	43 (82.7)	86 (83.5)	<.001
Fecal incontinence, n (%)					
Ever experienced	158 (52.7)	35 (68.6)	40 (76.9)	75 (72.8)	<.001
Currently experiencing	47 (15.7)	17 (33.3)	21 (40.4)	38 (36.9)	1
PF experience					
Number of unique PF, mean (SD)	–	2.2 (1.5)	2.8 (3.5)	–	NA
Currently active PF, n (%)	–	30 (58.8)	32 (61.5)	–	NA
Experience with PF recurrence/persistence, n (%)	–	24 (47.1)	24 (46.2)	–	NA
Diagnosing physician for CPF, n (%)					
Gastroenterologist	–	33 (64.7)	28 (53.8)	61 (59.2)	NA
Primary care physician	–	4 (7.8)	6 (11.5)	10 (9.7)	NA
Colorectal surgeon	–	13 (25.5)	17 (32.7)	30 (29.1)	NA
Other	–	1 (2.0)	1 (1.9)	2 (1.9)	NA
CPF diagnosis method, n (%)					
Physical examination with anesthesia	–	26 (51.0)	29 (55.8)	55 (53.4)	NA
Physical examination without anesthesia	–	19 (37.3)	22 (42.3)	41 (39.8)	NA
Endoanal ultrasound	–	13 (25.5)	13 (25.0)	26 (25.2)	NA
Endoscopy	–	18 (35.3)	17 (32.7)	35 (34.0)	NA
MRI	–	10 (19.6)	11 (21.2)	21 (20.4)	NA
Other	–	0 (0.0)	1 (1.9)	1 (1.0)	NA

CD, Crohn’s disease; CPF, Crohn’s perianal fistula; MRI, magnetic resonance imaging; NA, not applicable; non-PF CD, Crohn’s disease without perianal fistula; PF, perianal fistula; SD, standard deviation.

a May have received seton placement(s).

CD was classified by physicians as severe in more patients with CPF than non-PF CD (28.2% vs 13.0%; *P* < .001); more than half of the patients in each of the 3 cohorts (54%–64%) had moderate disease. Compared with the non-PF CD cohort, numerically more patients with CPF had experienced a recent CD flare, defined as sudden reactivation of symptoms (62% vs 49%), and had a significantly higher mean (standard deviation) rate of CD-related complications experienced at any time (9.5 [4.1] vs 5.8 [4.1]; *P* = .002). Patients with CPF were significantly more likely to be currently taking biologic therapies and immunomodulators than patients with non-PF CD (Table).

### Symptom Frequency and Severity

**Figure 1:**
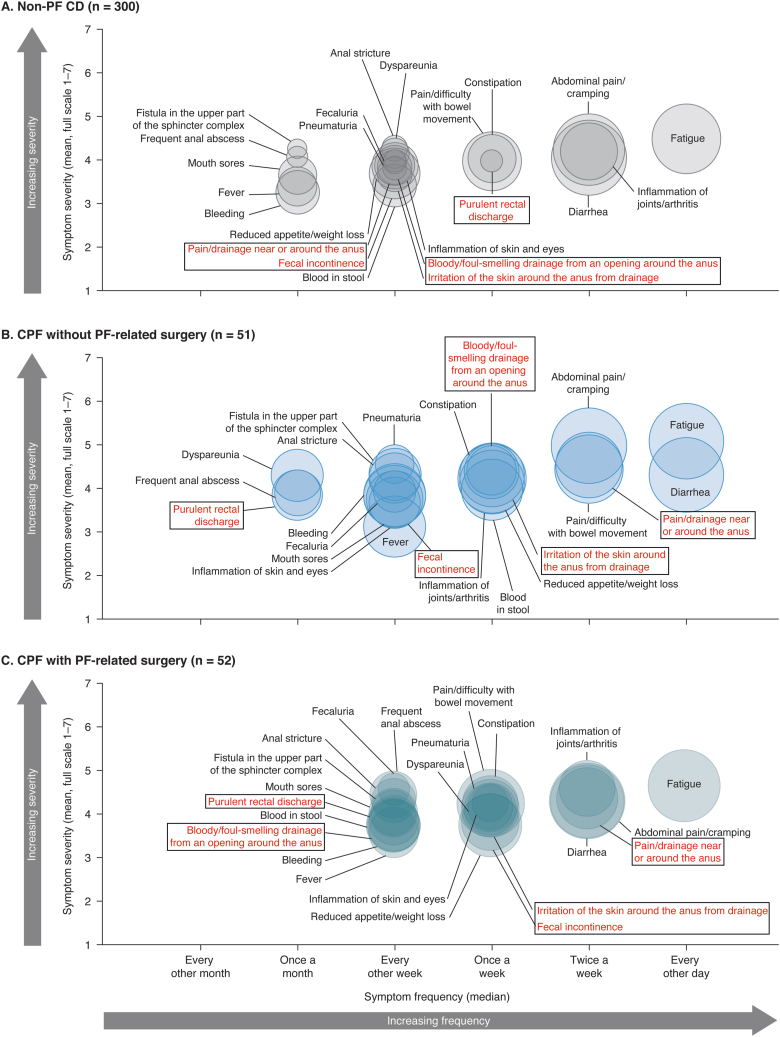
CD-specific symptom frequency and severity in the past 12 months for patients with (A) non-PF CD, (B) CPF without PF-related surgery, and (C) CPF with PF-related surgery. Red labels represent symptoms relating to anal leakage/incontinence. The larger the bubble size, the higher the sample size that experienced the symptom. When the median fell between 2 frequency values, the number was rounded up. CD, Crohn’s disease; CPF, Crohn’s perianal fistula; non-PF CD, Crohn’s disease without perianal fistula; PF, perianal fistula.

CD-specific symptom frequency and severity results in the past 12 months indicated a high symptom burden across cohorts (Figure 1). Fatigue was the most burdensome symptom reported by 87%–96% of patients in each cohort. Most patients reported experiencing fatigue every other day and the mean severity score ranged from 4.5 to 5.1 (symptom severity scale score range 1–7). The next most frequently reported symptoms across all cohorts were diarrhea, abdominal pain and cramping, pain/difficulty with bowel movement, and reduced appetite/weight loss (Figure A1). Fecal incontinence and leakage-related symptoms affected greater proportions of patients with CPF (with and without surgery), often with greater severity and frequency, than patients with non-PF CD (Figure 1).

### CD- and PF-related Procedures/Surgeries

Patients with CPF were significantly more likely than patients in the non-PF CD cohort to report having ever undergone ≥1 CD-related surgeries (79% vs 53%; *P* < .0001) and having ever experienced a surgical failure (defined as one that had an undesirable or unintended result). Twenty percent of patients with CPF experienced failure of ≥1 CD-related surgeries vs 9% of patients with non-PF CD (*P* = .0007).

**Figure 2:**
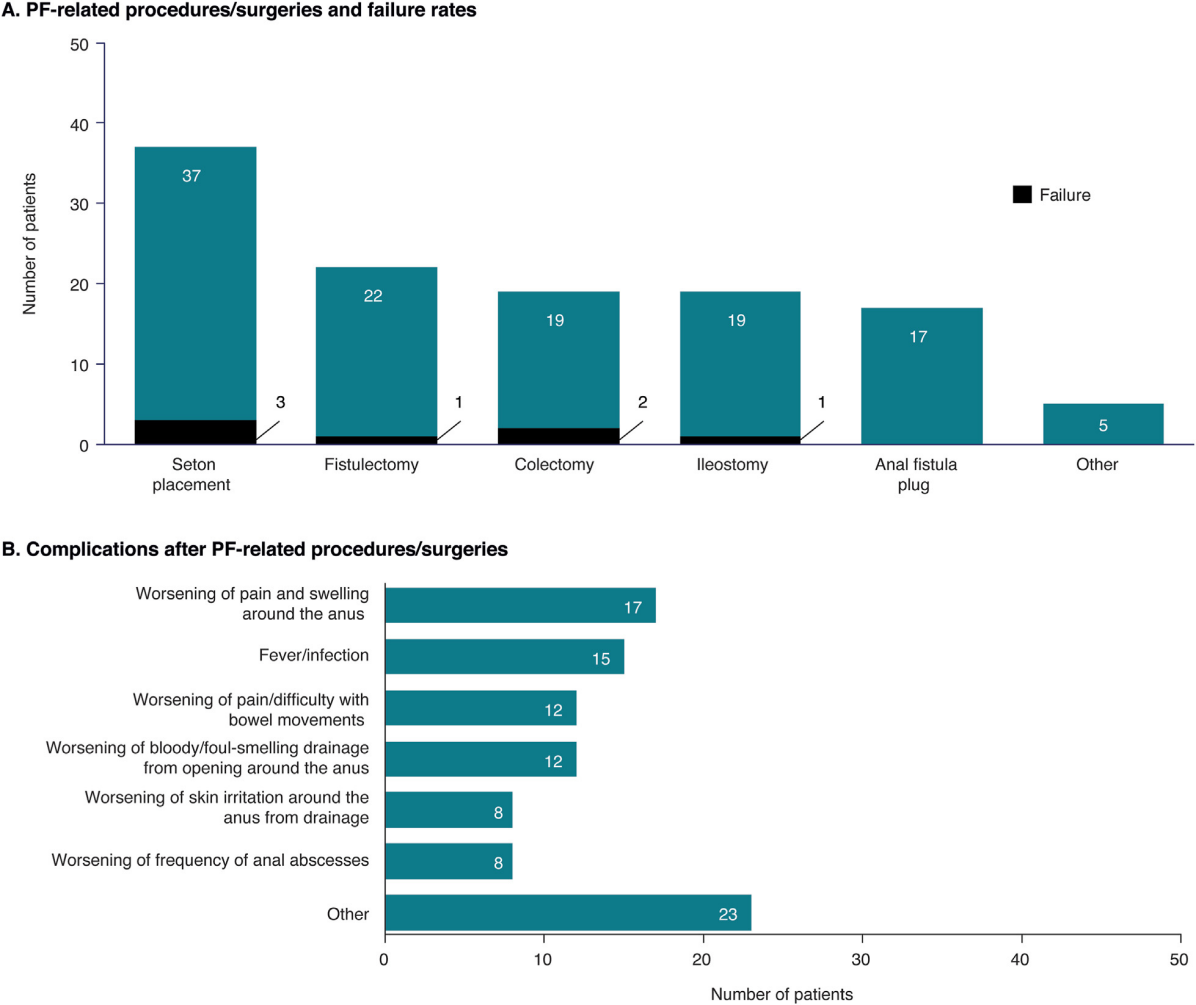
(A) Frequency of PF-related procedures/surgeries and respective failure rates at any time and (B) frequency of complications after PF-related procedures/surgeries in patients with CPF (cohort 3; n = 52). CPF, Crohn’s perianal fistulas; PF, perianal fistula.

Among the 52 patients in the CPF with surgery cohort, 33 (63%) reported undergoing ≥3 PF-related procedures/surgeries; seton placement was the most frequently reported procedure (Figure 2A). As well as treatment failure, post-surgical or procedural complications were common; the most frequently reported were worsening of pain and swelling around the anus (33%), and fever or infection (29%) (Figure 2B).

### Patient-reported HRQoL Outcomes

**Figure 3:**
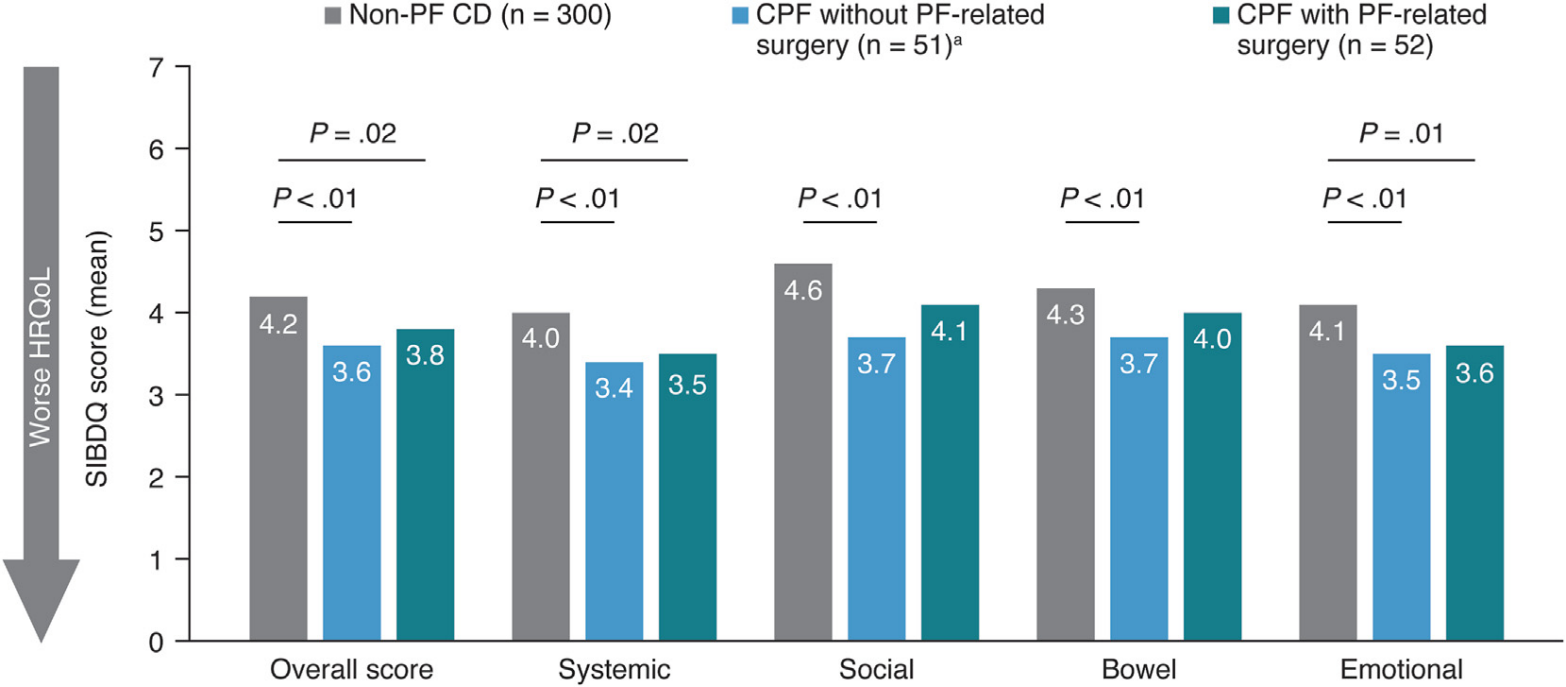
SIBDQ scores in patients with non-PF CD and patients with CPF with or without PF-related surgery. ^a^May have received seton placement(s). CPF, Crohn’s perianal fistula; HRQoL, health-related quality of life; non-PF CD, Crohn’s disease without perianal fistula; PF, perianal fistula; SIBDQ, Short Inflammatory Bowel Disease Questionnaire.

Overall mean SIBDQ scores were lowest (worst) for patients with CPF without surgery (3.6) and highest for patients with non-PF CD (4.2; *P* < .01). Significantly lower mean SIBDQ scores were also seen across all domains for the CPF without surgery cohort vs the non-PF CD cohort (all *P* < .01). The CPF with surgery cohort had significantly lower scores vs non-PF CD overall and for the Systemic Symptoms and Emotional Health domains (*P* = .02) (Figure 3).

SIBDQ scores were numerically lower for all patients with CPF, regardless of surgery (cohorts 2 and 3), than for patients with non-PF CD, even after controlling for patient demographics and other socioeconomic factors through multivariable analysis (Table A1). Paid employment, experiencing a CD flare, and not having a bachelor’s or master’s degree were also associated with negative impacts on SIBDQ score (*P* < .01).

**Figure 4:**
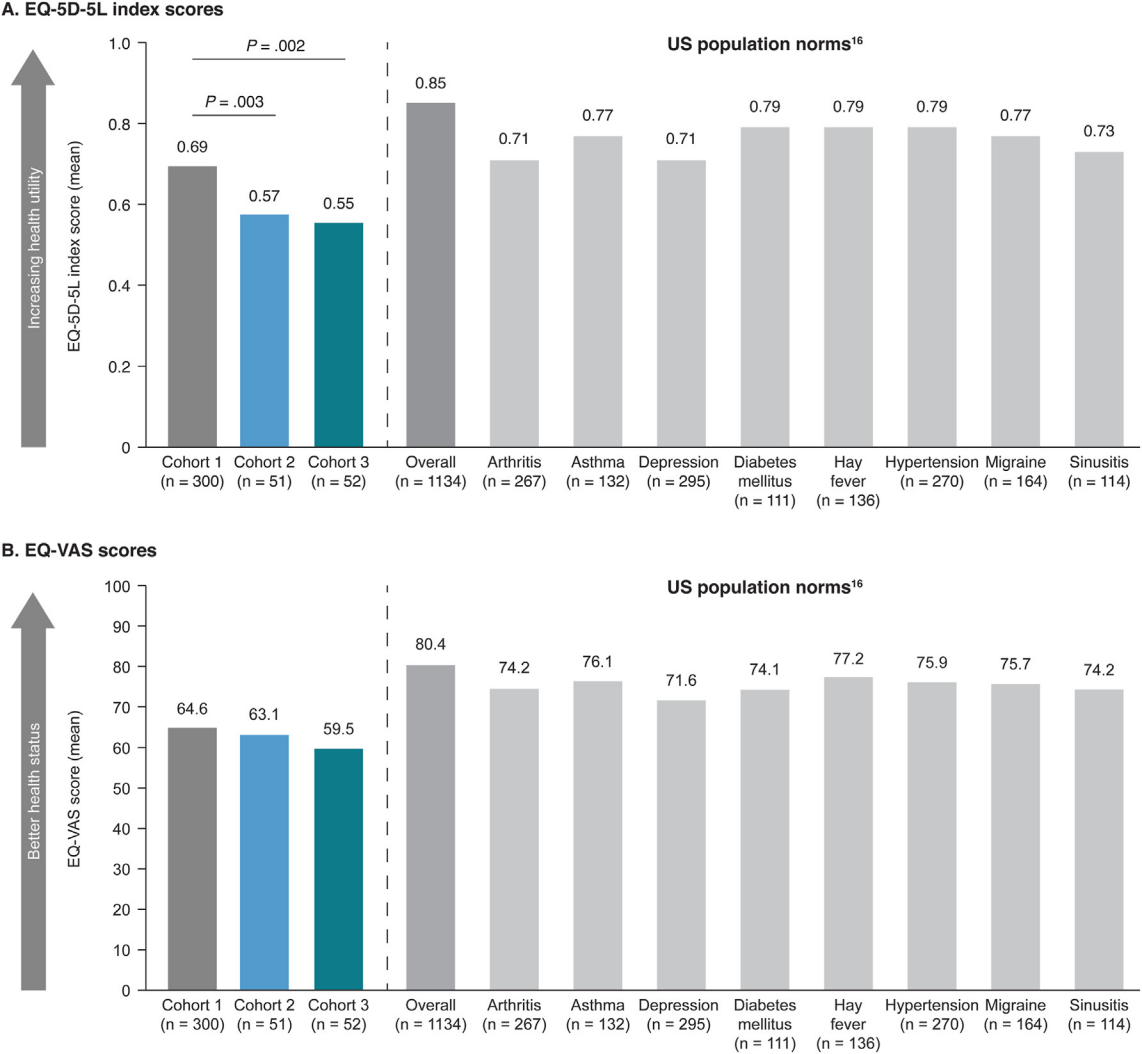
(A) EQ-5D-5L index scores and (B) EQ-VAS scores in patients with non-PF CD and patients with CPF with or without PF-related surgery, with US population norms for other disease states. CPF, Crohn’s perianal fistula; EQ-5D-5L, 5-level 5-dimension EuroQol questionnaire; EQ-VAS, EuroQol visual analog scale; non-PF CD, Crohn’s disease without perianal fistula; PF, perianal fistula.

Mean overall EQ-5D-5L index scores were also significantly lower (worse) for patients with CPF than for patients with non-PF CD (Figure 4A). Index scores were lower for all 3 cohorts compared with previously published data for the United States general population (0.85) and compared with other disease states within the United States population, including diabetes (0.79) and depression (0.71).^16^ Patients with CPF reported more severe impact on HRQoL compared with patients with non-PF CD across all EQ-5D-5L dimensions (Figure A2). Pain/discomfort and anxiety/depression were the 2 most severely affected dimensions across all 3 cohorts and significantly more patients with CPF reported severe problems with anxiety/depression than patients with non-PF CD.

In multivariable analyses adjusting for patient demographics and other socioeconomic factors, CPF was significantly associated with lower EQ-5D-5L index scores (worse health utility) compared with non-PF CD (*P* < .01) (Table A2). Being male and experiencing a recent CD flare were also associated with worse health utility, compared with being female and not experiencing a recent CD flare, respectively. Non-Hispanic White ethnicity was associated with better health utility vs other ethnic groups.

There were no statistically significant variations in mean EQ visual analog scale scores across cohorts and visual analog scale scores were worse than those for a range of diseases within the United States population, including diabetes and depression (Figure 4B).^16^

### Fecal Incontinence

**Figure 5:**
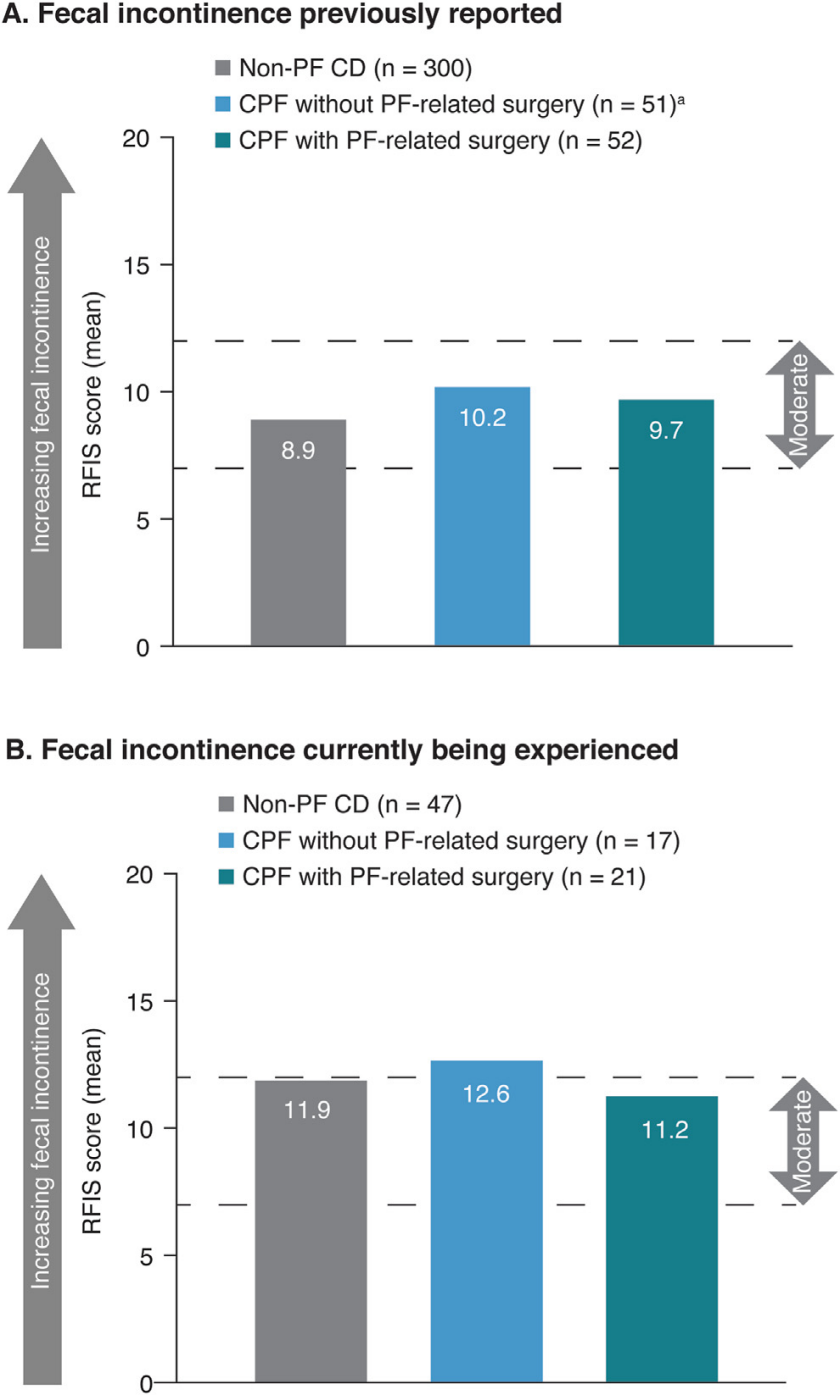
RFIS scores in (A) patients with non-PF CD and patients with CPF with or without PF-related surgery who previously reported fecal incontinence and (B) those currently experiencing fecal incontinence. ^a^May have received seton placement(s). CPF, Crohn’s perianal fistula; non-PF CD, Crohn’s disease without perianal fistula; PF, perianal fistula; RFIS, Revised Faecal Incontinence Scale.

Across all cohorts, 58% of patients experienced fecal incontinence and therefore completed the RFIS and FIQL (n = 158, 35, and 40 in the non-PF CD, CPF without surgery, and CPF with surgery cohorts, respectively). Patients who had reported experience of fecal incontinence had similarly moderate (ie, score 7–12) mean RFIS scores across all cohorts; however, scores were directionally higher in the CPF without surgery cohort (mean 10.2) and CPF with surgery cohort (mean 9.7) than in the non-PF CD cohort (mean 8.9) (Figure 5A). Patients currently experiencing fecal incontinence had higher mean RFIS scores across all 3 cohorts than those who previously experienced fecal incontinence, and again, the CPF without surgery cohort showed directionally higher RFIS scores (mean 12.6) compared with the non-PF CD cohort (mean 11.9) and CPF with surgery cohort (mean 11.2) (Figure 5B).

Multivariable analysis was conducted to assess the association of patient characteristics with RFIS. Patients with CPF had a higher RFIS score compared with those with non-PF CD, even after controlling for patient demographics and other socioeconomic factors, although the difference was not statistically significant (Table A3). Experiencing a recent CD flare was also associated with a higher RFIS score, and non-Hispanic White ethnicity was associated with lower RFIS scores vs other ethnic groups (both *P* ≤ .01).

Patients with CPF (with and without surgery) also had numerically lower (worse) FIQL scores consistently across all 4 domains compared with patients with non-PF CD (Figure A3).

## Discussion

This study compared the HRQoL, experiences, and perceptions of patients with CPF and those with non-PF CD, using validated patient-reported outcomes measures and general questionnaires. For patients with CPF, these outcomes were also compared between those with PF-related surgery and those without. Our results revealed that patients with CPF had a higher disease and HRQoL burden compared with patients with non-PF CD, regardless of whether they had PF-related surgery, and a higher proportion received medical and surgical interventions.

Compared with patients with non-PF CD, patients with CPF were significantly more likely to be diagnosed as having severe CD (28% vs 13%) and to have experienced higher rates of CD-related complications (84% experienced >5 complications vs 41%). This aligns with the perception that PFs are a marker of more severe disease^6^ and supports the idea that patients with CPF may require unique clinical considerations to manage their disease optimally. Patients with CPF were significantly more likely to be currently receiving biologic therapies and immunomodulators than patients with non-PF CD (biologics: 58% vs 43%; immunosuppressants: 23% vs 15%, respectively). Although not necessarily indicative of the rates of biologic escalation in patients with CPF, these results appear to reflect US clinical guidelines for the management of CPF. Surgical burden was also high among patients with CPF, particularly when coupled with the rate of surgical failure (20%). Further, more than half of patients with CPF who had undergone PF-related surgery reported undergoing ≥3 PF-related surgeries; among those, the rates of surgical failure were lower than for CD-related surgeries (5%–11% depending on the procedure), but post-surgical or procedural complications were common.

Previous studies have shown that patients with CD who develop PF commonly experience symptoms such as fecal incontinence, anal pain, and discharge, which significantly affect HRQoL.^13^^,^^19^ One study found that patients with inflammatory bowel disease and perianal disease experienced worse physical functioning, fatigue, emotional well-being, and social functioning compared with patients who did not have perianal disease.^19^ In another qualitative assessment in patients with CPF, Adegbola et al^13^ identified fistula-related pain, discharge, and fatigue as key subthemes within the overarching theme of symptom burden. Of note, participants often reported considerable fatigue attributed to their constant experience of anal fistula pain and not their underlying CD.^13^ In the current study, fatigue was the most burdensome symptom, reported with the highest frequency and severity across cohorts. Over half (58%) of patients in this study reported fecal incontinence and patients with CPF were often found to have more frequent and more severe fecal incontinence and leakage-related symptoms than those with non-PF CD. This is reflected in the lower (worse) FIQL scores for patients with CPF compared with those with non-PF CD. However, caution should be given when interpreting these data because fecal incontinence may be related to CD alone, rather than to PF discharge or surgical interventions.

Although CD itself affects HRQoL, patients with CPF consistently reported worse HRQoL than patients with non-PF CD, both in general and disease-specific assessments, possibly reflecting the greater frequency and severity of symptoms in patients with CPF. These patients reported higher (worse) scores for pain/discomfort and anxiety/depression, but no difference was noted for self-care, mobility, and usual activities, as measured using the EQ-5D. Similarly, patients had worse scores for the SIBDQ. Consistent with reported symptoms, patients with CPF experiencing current fecal incontinence had worse RFIS and FIQL scores than patients with non-PF CD, indicating that the burden of fecal incontinence had a greater negative impact on HRQoL for patients with CPF. Patients currently experiencing fecal incontinence had worse RFIS scores across all 3 cohorts than those who previously experienced fecal incontinence. The CPF-associated disease burden persisted even after controlling for confounding variables across patient demographics and socioeconomic factors in multivariable analyses of EQ-5D, SIBDQ, and RFIS scores. Further comparisons of patients with CPF showed only minimal differences in symptom scores or HRQoL scores between patients who had or had not undergone PF-related surgery, suggesting that surgical intervention may not provide sustained improvements in symptoms or HRQoL. Indeed, surgeries may even have a negative impact on physical and emotional HRQoL, as suggested by the findings of Adegbola et al.^13^ Thus, there is an unmet need for better treatment(s) to manage PF that will improve patient outcomes and HRQoL.

This study has some limitations, including recall bias and patients’ understanding of medical terms. These risks were partly mitigated by limiting the recall period to 12 months and conducting cognitive interviews before the web-enabled questionnaire to ensure patients’ understanding of the questions was as intended. There is also a risk of selection bias as patients with more severe disease may be more likely to respond to the questionnaire. Additionally, by including only a convenience sample of patients in the United States, the results may not be representative of patients with CD outside the study and in other countries. Furthermore, patients enrolled in this study were not assessed by physicians, which could lead to errors in eligibility for each cohort. Finally, self-reporting of symptoms is subjective and prone to inaccuracies. In the case of a CD flare-up, patients may report reactivation of symptoms without a confirmed presence of inflammation. This may also be true for self-reporting of surgeries, in which patients may be inaccurate in their understanding of the surgery types experienced.

## Conclusion

The findings from this study indicate that patients with CPF experience a substantial HRQoL burden, reflecting the impact of symptoms and medical or surgical interventions. For many of the aspects of disease assessed, the burden was significantly greater in patients with CPF compared with patients with CD without PF. These results may be useful in helping to tailor comprehensive care strategies to better support patients with CPF and improve their overall quality of life.

## Supplementary Materials

Supplementary Data Content
