# Supplementary material for: Observational Burden of Illness Study in Patients With Crohn’s Disease With and Without Perianal Fistulas in the United States
Source: Gastro Hep Adv. 2023 Aug 24;2(8):1066–76. doi: 10.1016/j.gastha.2023.08.011 (PMC11307624; doi:10.1016/j.gastha.2023.08.011)
Supplement: Supplementary Data Content [file mmc1.docx]

# SUPPLEMENTARY DATA CONTENT

Supplement to: ‘Observational Burden of Illness Study in Patients with Crohn’s Disease With and Without Perianal Fistulas in the USA’.

Jeanne Jiang, Susan E. Cazzetta, Amod Athavale, Maja Kuharic, Tao Fan, Abby Silber, Vijay Abilash, Nandini Hadker, Emily Sharpe, and Pradeep P. Nazarey.

# Contents:

## Supplementary Figure 1. Symptom frequency and severity in the past 12 months for the 5 most reported symptoms in patients with non-PF CD and patients with CPF with or without PF-related surgery.

## Supplementary Figure 2. Patient-reported impact across EQ-5D-5L dimensions in patients with non-PF CD and patients with CPF with or without PF-related surgery.

## Supplementary Figure 3 FIQL questionnaire scores in patients with non-PF CD and patients with CPF with or without PF-related surgery.

## Supplementary Table 1. Multivariable Analysis of SIBDQ Scores in Patients With Non-PF CD and Patients With CPF With or Without PF-related Surgery After Controlling for Patient Demographics and Socioeconomic Factors.

## **Supplementary Table 2.** Multivariable Analysis of EQ-5D-5L Index Scores in Patients With Non-PF CD and Patients With CPF With or Without PF-related Surgery After Controlling for Patient Demographics and Socioeconomic Factors.

## **Supplementary Table 3.** Multivariable Analysis of RFIS Scores in Patients With Non-PF CD and Patients With CPF With or Without PF-related Surgery After Controlling for Patient Demographics and Socioeconomic Factors.

## Supplementary Figure 1. Symptom frequency and severity in the past 12 months for the 5 most reported symptoms in patients with non-PF CD and patients with CPF with or without PF-related surgery.


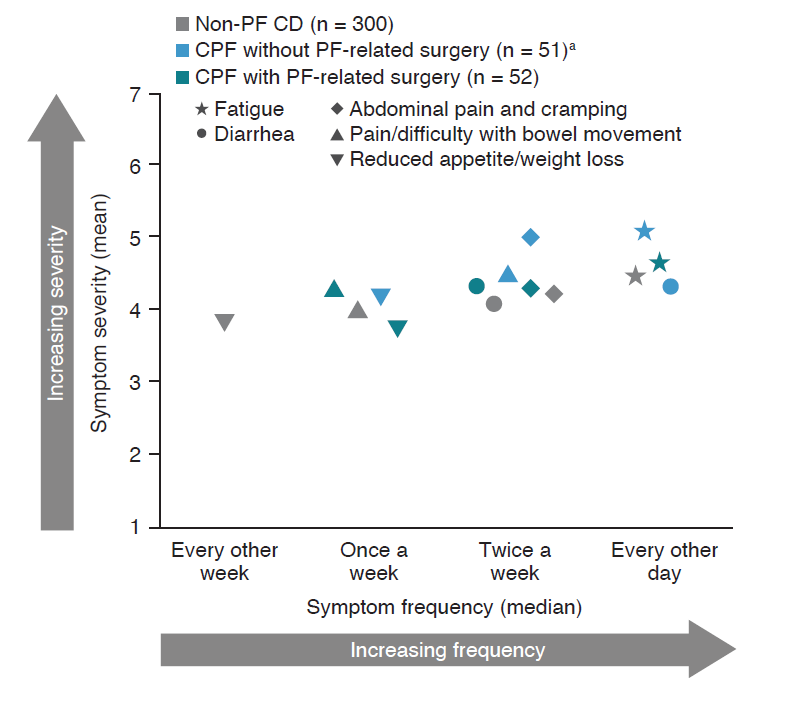


CPF, Crohn’s perianal fistula; non-PF CD, Crohn’s disease without perianal fistula; PF, perianal fistula.

## Supplementary Figure 2. Patient-reported impact across EQ-5D-5L dimensions in patients with non-PF CD and patients with CPF with or without PF-related surgery.


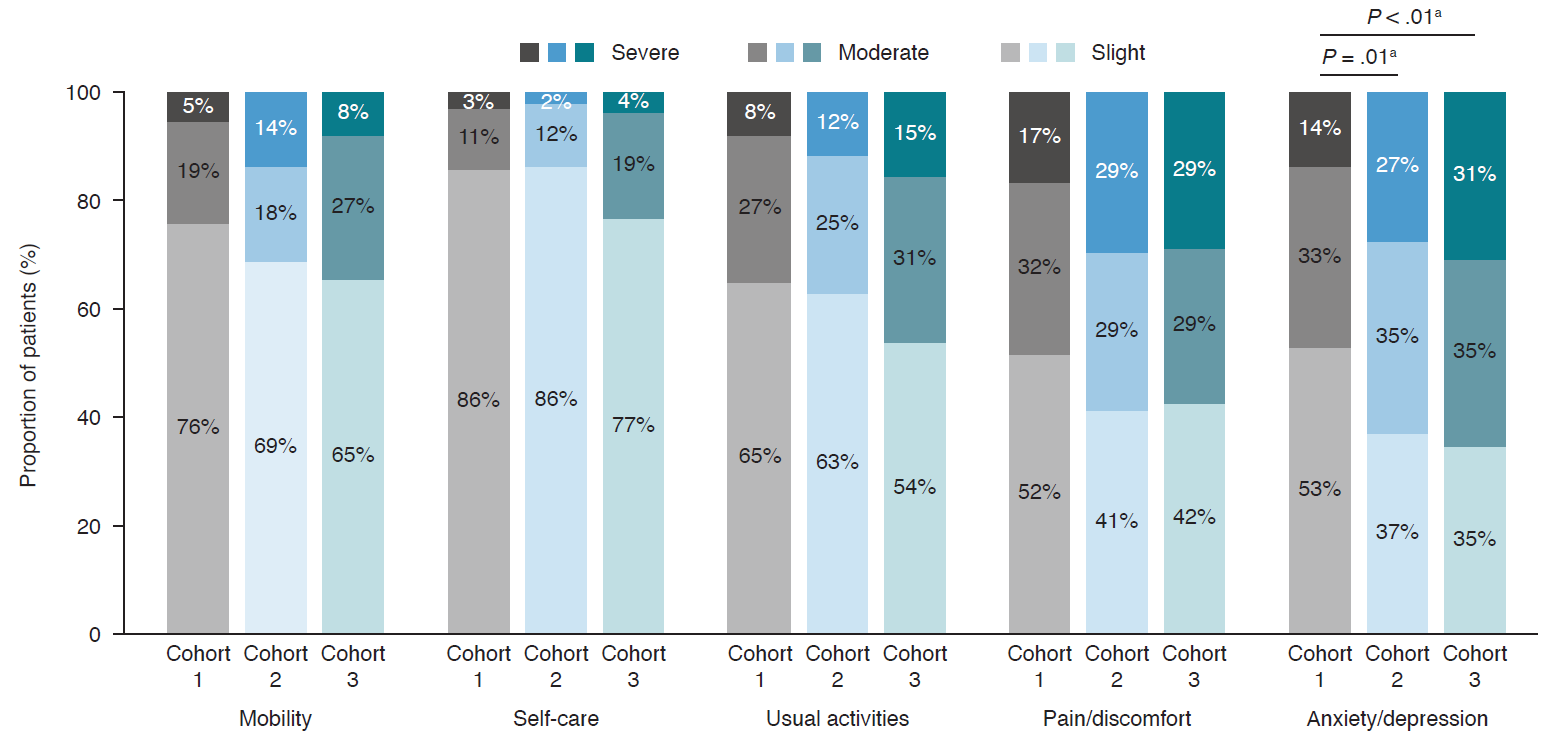


‘Severe’ includes patients reporting problems as severe and extremely severe/unable to do; ‘Moderate’ includes patients reporting problems as moderate; ‘Slight’ includes patients reporting no and slight problems.

Cohort 1, patients with non-PF CD; cohort 2, patients with CPF without surgery; cohort 3, patients with CPF with surgery.

^a^Statistical significance testing for the ‘Severe’ category only.

CPF, Crohn’s perianal fistula; non-PF CD, Crohn’s disease without perianal fistula; EQ-5D, 5-dimension EuroQol questionnaire; PF, perianal fistula.

## Supplementary Figure 3. FIQL questionnaire scores in patients with non-PF CD and patients with CPF with or without PF-related surgery.


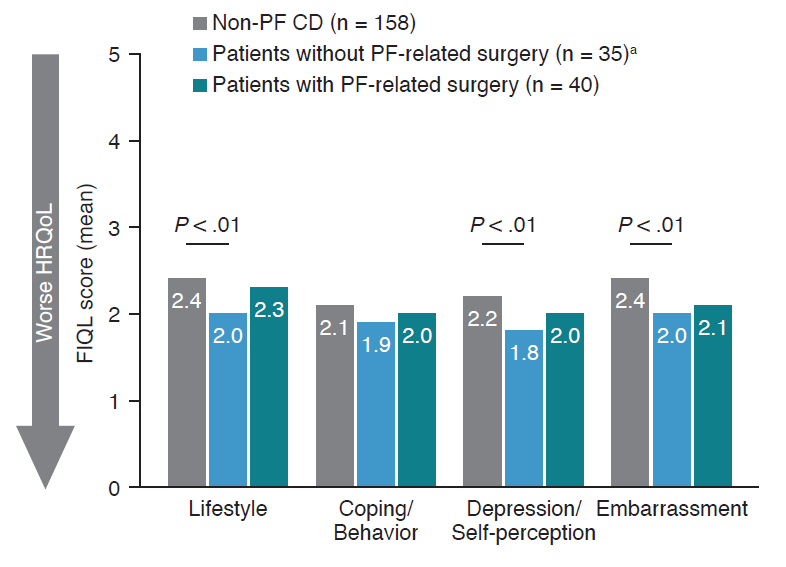


^a^These patients may have received seton placement(s).

The FIQL scale comprises four domains (Lifestyle, Coping/Behavior, Depression/Self perception, Embarrassment). The scale has a range of 0–5, with lower scores indicating worse quality of life.

CPF, Crohn’s disease with perianal fistulas; FIQL, Fecal Incontinence Quality of Life; HRQoL, health-related quality of life; non-PF CD, Crohn’s disease without perianal fistulas; PF, perianal fistula.

## Supplementary Table 1. Multivariable analysis of SIBDQ scores in patients with non-PF CD and patients with CPF with or without PF-related surgery after controlling for patient demographics and socioeconomic factors.

|  | Estimate | Standard error | t value | *P* value |
| --- | --- | --- | --- | --- |
| Intercept | 3.56 | 0.34 | 10.34 | <.01 |
| CPF *(vs non-PF CD)* | −0.21 | 0.12 | −1.81 | .07 |
| Male *(vs female)* | −0.14 | 0.12 | −1.21 | .23 |
| Employed for wages *(vs not employed)* | −0.44 | 0.13 | −3.40 | <.01 |
| Experienced a CD flare *(vs did not experience a flare)* | −0.83 | 0.10 | −8.06 | <.01 |
| Time since CD diagnosis, years | 0.02 | 0.01 | 3.67 | <.01 |
| Household income ≤US$79K *(vs household income >US$80K)* | −0.12 | 0.12 | −1.02 | .31 |
| Private healthcare insurance^a^ *(vs not on private insurance/on other insurance)*^b^ | 0.07 | 0.12 | 0.56 | .58 |
| No bachelor’s or master’s degree *(vs having a bachelor’s or master’s degree)* | −0.36 | 0.11 | −3.23 | <.01 |
| Single^c^ *(vs married)* | 0.17 | 0.12 | 1.48 | .14 |
| Non-Hispanic White *(vs other races/ethnicities)* | 0.30 | 0.13 | 2.37 | .02 |
| Not diagnosed with chronic fatigue *(vs diagnosed with chronic fatigue)* | 0.59 | 0.20 | 3.03 | <.01 |
| Current age, years | 0.01 | <0.01 | 2.84 | .01 |

Adjusted *R*^2^ = 0.37. The dependent variable was the SIBDQ score (range 1–7; 1 = poor HRQoL, 7 = optimum HRQoL).

^a^Includes self-purchased and/or employer-provided or commercial insurance.

^b^Excludes self-purchased and/or employer-provided or commercial insurance; includes Medicare, Medicaid, other government insurance or no insurance.

^c^Includes divorced, widowed, or separated.

CD, Crohn’s disease; CPF, Crohn’s perianal fistula; HRQoL, health-related quality of life; non-PF CD, Crohn’s disease without perianal fistula; PF, perianal fistula; SIBDQ, Short Inflammatory Bowel Disease Questionnaire.

## **Supplementary Table 2.** Multivariable analysis of EQ-5D-5L index scores in patients with non-PF CD and patients with CPF with or without PF-related surgery after controlling for patient demographics and socioeconomic factors.

|  | Estimate | Standard error | t value | *P* value |
| --- | --- | --- | --- | --- |
| Intercept | 0.61 | 0.06 | 9.45 | <.01 |
| CPF *(vs non-PF CD)* | **−0.08** | **0.03** | **−3.12** | <.01 |
| Male *(vs female)* | −0.09 | 0.03 | −3.28 | <.01 |
| Employed for wages *(vs not employed)* | −0.02 | 0.03 | −0.82 | .41 |
| Experienced a CD flare *(vs did not experience a flare)* | −0.10 | 0.02 | −4.10 | <.01 |
| Time since CD diagnosis, years | <0.01 | <0.01 | 1.68 | .09 |
| Household income ≤US$79K *(vs household income >US$80K)* | −0.01 | 0.03 | −0.23 | .82 |
| Private healthcare insurance^a^ *(vs not on private insurance/on other insurance)*^b^ | 0.05 | 0.03 | 1.95 | .05 |
| No bachelor’s or master’s degree *(vs having a bachelor’s or master’s degree)* | −0.02 | 0.03 | −0.63 | .53 |
| Single^c^ *(vs married)* | 0.04 | 0.03 | 1.53 | .13 |
| Non-Hispanic White *(vs other races/ethnicities)* | 0.09 | 0.03 | 3.04 | <.01 |
| Current age, years | <0.01 | <0.01 | 1.19 | .24 |

Adjusted *R*^2^ = 0.20. The dependent variable was the EQ-5D-5L index score.

^a^Includes self-purchased and/or employer-provided or commercial insurance.

^b^Excludes self-purchased and/or employer-provided or commercial insurance; includes Medicare, Medicaid, other government insurance or no insurance.

^c^Includes divorced, widowed, or separated.

CD, Crohn’s disease; CPF, Crohn’s perianal fistula; EQ-5D, 5-dimension EuroQol questionnaire; HRQoL, health-related quality of life; non-PF CD, Crohn’s disease without perianal fistula; PF, perianal fistula.

## **Supplementary Table 3.** Multivariable analysis of RFIS scores in patients with non-PF CD and patients with CPF with or without PF-related surgery after controlling for patient demographics and socioeconomic factors.

|  | Estimate | Standard error | t value | *P* value |
| --- | --- | --- | --- | --- |
| Intercept | 7.57 | 1.50 | 5.06 | <.01 |
| Patient with CPF *(vs non-PF CD)* | 0.49 | 0.58 | 0.86 | .39 |
| Male *(vs female)* | 1.20 | 0.62 | 1.95 | .05 |
| Employed for wages *(vs not employed)* | 0.35 | 0.72 | 0.49 | .63 |
| Experienced a CD flare *(vs did not experience a flare)* | 1.92 | 0.55 | 3.49 | <.01 |
| Time since CD diagnosis, years | −0.01 | 0.03 | −0.41 | .68 |
| Household income ≤US$79K *(vs household income >US$80K)* | 1.26 | 0.62 | 2.03 | .04 |
| Private healthcare insurance^a^ *(vs not on private insurance/on other insurance)*^b^ | −0.12 | 0.61 | −0.19 | .85 |
| No bachelor’s or master’s degree *(vs having a bachelor’s or master’s degree)* | 0.31 | 0.60 | 0.51 | .61 |
| Single^c^ *(vs married)* | −0.94 | 0.60 | −1.56 | .12 |
| Non-Hispanic White *(vs other races/ethnicities)* | −1.85 | 0.66 | −2.80 | .01 |
| Current age, years | 0.02 | 0.02 | 0.79 | .43 |

Adjusted *R*^2^ = 0.14. The dependent variable was the RFIS score (range 0–20; <4 = no/very mild fecal incontinence, 4–6 = mild, 7–12 = moderate, ≥13 = severe).

^a^Includes self-purchased and/or employer-provided or commercial insurance.

^b^Excludes self-purchased and/or employer-provided or commercial insurance; includes Medicare, Medicaid, other government insurance or no insurance.

^c^Includes divorced, widowed, or separated.

CD, Crohn’s disease; CPF, Crohn’s perianal fistula; HRQoL, health-related quality of life; non-PF CD, Crohn’s disease without perianal fistula; PF, perianal fistula; RFIS, Revised Faecal Incontinence Scale.
